# Simultaneous Determination and Quantification of Triterpene Saponins from *Camellia sinensis* Seeds Using UPLC-PDA-QTOF-MS/MS

**DOI:** 10.3390/molecules24203794

**Published:** 2019-10-22

**Authors:** Xuejin Wu, Lingyan Jia, Jiafan Wu, Yawen Liu, Hyunuk Kang, Xiaobo Liu, Pan Li, Puming He, Youying Tu, Bo Li

**Affiliations:** Department of Tea Science, Zhejiang University, 866 Yuhangtang Road, Hangzhou 310058, China

**Keywords:** saponin, *Camellia sinensis* seed, UPLC-PDA-QTOF-MS/MS, identification, quantitation, vanillin-sulfuric acid

## Abstract

Saponins in the *Camellia sinensis* seeds have a broad spectrum of biological properties and application potentials. However, up to now, no chromatographic methods have been developed to provide full fingerprinting and quality assurance for these saponins. This research aimed to develop a novel method to tentatively identify and quantify saponins in *C. sinensis* seeds by ultra-high-performance liquid chromatography coupled with photo-diode array detector and quadrupole time-of-flight mass spectrometry (UPLC-PDA-QTOF-MS/MS), and compare it with the classic vanillin-sulfuric acid assay. Fifty-one triterpene saponins, including six potentially new compounds, were simultaneously detected by UPLC-PDA-MS/MS, and their chemical structures were speculated according to the retention behavior and fragmentation pattern. The total saponin content in the crude extract and the purified saponin fraction of *C. sinensis* seeds were quantified to be 19.57 ± 0.05% (wt %) and 41.68 ± 0.09% (wt %) respectively by UPLC-PDA at 210 nm, while the corresponding values were determined to be 43.11 ± 3.17% (wt %) and 56.60 ± 5.79% (wt %) respectively by the vanillin-sulfuric acid assay. The developed UPLC-PDA -MS/MS method could determine specified saponins, and is more reliable for quantifying the *C. sinensis* seed saponins than the classic spectrophotometric method. It is of great significance for the future investigations and applications of these saponins.

## 1. Introduction

*Camellia sinensis* (L.) O. Kuntze, belonging to the family of Theaceae, is a woody perennial tree with great economic importance [1]. Tea manufactured from the leaves of *C. sinensis* is second in popularity only to water as a beverage in the world, and possesses many benefits to human health [2]. In addition to tea leaves, the seeds of *C. sinensis* contain a large number of bioactive constituents including saponins, flavonoids, unsaturated fatty acids, and polysaccharides, and are considered as a valuable resource for food, agriculture, pharmaceuticals, aquaculture, and the cosmetic industry [3,4,5,6,7].

Oleanane-type triterpene saponins were determined as one of the major bioactive ingredients in *C. sinensis* seeds. So far, more than 50 saponin compounds have been isolated and identified from the seeds of *C. sinensis* cultivated in China, Japan, Sri Lanka, and India [8]. Pharmacological studies indicated that these saponins could protect gastric mucosa of rats induced by ethanol or indomethacin [9], inhibit gastric emptying and alcohol absorption in mice [10], suppress ascites cancer S180 cells, myelocytic leukemia K562 cells, promyelocytic leukemia HL-60 cells, cisplatin-resistant ovarian cancer OVCAR-3 and A2780/CP70 cells in vitro and in vivo [11,12], block inflammatory pathways composed of AKT, IKK, and NF-κB in macrophages stimulated by fluorescein isothiocyanate-dextran, sodium nitroprusside and lipopolysaccharide [13], and have a wide spectrum of anti-bacterial and anti-fungal activities [14,15,16]. In addition, *C. sinensis* seed saponins could promote uptake of Cd by *Amaranthus caudatus*, and were more efficient than ethylenediaminetetracetic acid (EDTA) [17].

Although *C. sinensis* seed saponins have gotten increasing attention due to a broad spectrum of biological properties and application potentials, the studies on the analysis methods for these compounds are still limited. These saponins occur as a multicomponent mixture with similar structures and polarities, which causes a challenge for their isolation and determination, and thus limits in-depth investigation into their bioactivities and applications. Spectrophotometric and liquid chromatographic methods are usually employed to determine saponins from various plant materials [18]. The vanillin-sulfuric acid assay is the most commonly selected spectrophotometric method for saponin quantification since it is simple, fast and inexpensive to operate [19,20]. However, this method can only measure the total saponin content, and its color reaction is not specific [21]. Hyphenated techniques coupling thin-layer chromatography (TLC), high-performance liquid chromatography (HPLC) and ultra-performance liquid chromatography (UPLC) with an ultraviolet detector (UV), evaporative light scattering detector (ELSD) and mass spectrometry (MS) have been applied to characterize and quantify plant saponins [22,23,24,25,26]. Nevertheless, no chromatographic methods have been developed to provide full fingerprinting and quality assurance for *C. sinensis* seed saponins up to now. The major difficulties are providing effective separation and identification without enough saponin standards.

The aim of the present study was to develop an effective ultra-high-performance liquid chromatography coupled with photo-diode array detector and mass spectrometry (UPLC-PDA-MS/MS) method for the simultaneous detection and quantification of *C. sinensis* seed saponins. Compound identification was performed based on the MS/MS data, predication of polarity and structure information of all reported saponins from the seeds of the genus *Camellia*. In addition, this chromatographic method and vanillin-sulfuric acid assay were compared to evaluate their reliability for quantifying *C. sinensis* seed saponins.

## 2. Results and Discussion

### 2.1. Optimization of UPLC-PDA-QTOF-MS Chromatography

**Figure 1:**
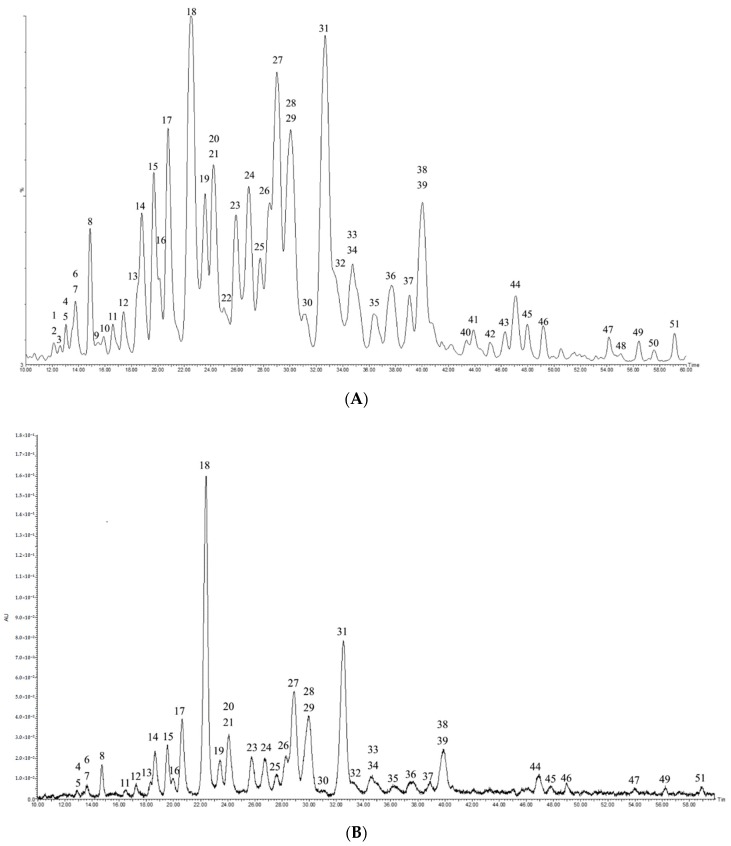

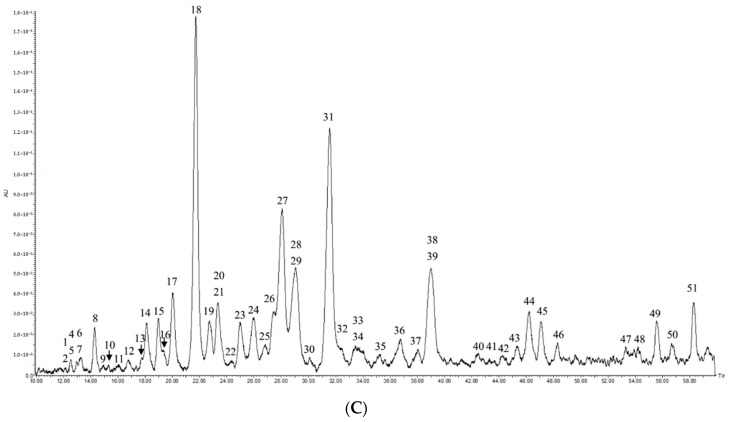
Saponins from the seeds of *Camellia sinensis* detected by ultra-high-performance liquid chromatography coupled with photo-diode array detector and quadrupole time-of-flight mass spectrometry (UPLC-PDA-QTOF-MS). (**A**) UPLC/MS total ion chromatogram of the crude extract of *C. sinensis* seeds (2.0 mg/mL) in negative ion mode. (**B**) UPLC-PDA chromatogram of the crude extract of *C. sinensis* seeds (2.0 mg/mL) at 210 nm. (**C**) UPLC-PDA chromatogram of the total saponin fraction of *C. sinensis* seeds (2.0 mg/mL) at 210 nm.

Ultra-high-performance liquid chromatography coupled with photo-diode array detector and quadrupole time-of-flight mass spectrometry (UPLC-PDA-QTOF-MS) was used to detect the saponins in the crude extract and total saponin fraction of *C. sinensis* seeds in this work. The crude extract was obtained under reflux with 70% methanol at 70 °C, and then was purified by successive extraction with different polar solvents (petroleum ether, ethyl acetate, and 1-butanol) and D101 column chromatography to yield the total saponins. This procedure is one of the classical methods for the extraction and purification of *Camellia* seed saponins as reviewed by Guo et al. recently [8]. The chromatographic conditions were optimized to achieve effective separation, symmetric peak shape and short run time. Three chromatographic columns, namely the Welch Ultimate UHPLC XB-C_18_ (1.8 μm, 100 mm × 2.1 mm i.d.), Waters ACQUITY UPLC BEH C_18_ (1.7 µm, 100 mm × 2.1 mm i.d.) and Waters ACQUITY UPLC HSS T3 column (1.8 μm, 150 mm × 2.1 mm i.d.) were pretested, and the best separation efficiency was obtained with the HSS T3 column. Acetonitrile was selected as the mobile phase due to its much lower absorption at lower UV wavelengths, improved separation, and reduced column back pressure compared with methanol [27]. The addition of formic acid in the mobile phase improved the peak shape, sensitivity, and retention time of individual saponins, which was consistent with the previous report [28]. Under the developed chromatographic condition, 51 saponins were simultaneously detected from the crude extract of *C. sinensis* seeds (2.0 mg/mL) in negative ion mode (Figure 1A), and 40 corresponding peaks were obviously observed under a PDA detector at a wavelength of 210 nm (Figure 1B). For the purified total saponin fraction (2.0 mg/mL), the UPLC/MS total ion chromatogram was similar to that of the crude extract (data not shown), and 50 saponins were detected at 210 nm (Figure 1C). The majority of saponins of *C. sinensis* seeds possess no chromophores, and could be detected at the non-specific ultraviolet wavelengths around 210 nm. This structure feature led to lower detection sensitivity under the ultraviolet (UV) detector compared with MS, and explained that some minor peaks were not observed in the UV chromatogram of the samples. For the purified total saponin fraction, the peaks from the UV and MS total ion current chromatograms were almost all corresponding with each other except for peak 3, indicating UV detector is more applicable for detecting *C. sinensis* seed saponins at higher concentrations.

### 2.2. Compound Characterization of Saponins by UPLC-QTOF-MS/MS

In our system, the signal intensity of the saponins obtained in the negative ionization mode was superior to that in the positive ionization mode, which was consistent with the previous studies [24]. In the precursor ion full-scan spectra, the 51 saponin peaks were detected with parent ions [M – H]^−^ at *m/z* 1157.5841–1315.6046 (Table 1). Among these compounds, eleven peaks have unique molecular ions [M – H]^−^ at *m/z* 1157.5841 (peak 38), *m/z* 1185.5774 (peak 45), *m/z* 1189.5742 (peak 5), *m/z* 1215.5892 (peak 37), *m/z* 1219.5854 (peak 3), *m/z* 1243.5835 (peak 46), *m/z* 1257.5991 (peak 43), *m/z* 1269.6005 (peak 51), *m/z* 1299.6105 (peak 50), *m/z* 1303.6111 (peak 21), and *m/z* 1315.6111 (peak 42), respectively. In addition, thirteen groups with similar molecular weights were found at *m/z* 1171.5630–1171.5670 (peaks 7, 28), *m/z* 1173.5783–1173.5809 (peaks 15, 19), *m/z* 1187.5587–1187.5953 (peaks 4, 8, 36, 40), *m/z* 1201.5759–1201.5779 (peaks 24, 32), *m/z* 1217.5686–1217.5693 (peaks 1, 6, 10), *m/z* 1229.5699–1229.5725 (peaks 2, 13, 18, 27, 31), *m/z* 1231.5838–1231.5873 (peaks 11, 14, 22), *m/z* 1259.5807–1259.5844 (peaks 12, 16, 20, 29, 33), *m/z* 1261.5947–1261.5989 (peaks 9, 25), *m/z* 1271.5805–1271.6146 (peaks 30, 39, 49), *m/z* 1273.5938–1273.5993 (peaks 17, 23, 34, 41), *m/z* 1285.5945–1285.5987 (peaks 44, 47), and *m/z* 1301.5922–1301.5942 (peaks 26, 35, 48).

Further identification was conducted based on the MS/MS spectra and fragmentation pathways, referring to previous reports on the saponins isolated from *C. sinensis* seeds [8]. First of all, the eleven peaks with unique ion [M – H]^−^ were tentatively identified. Peak 3 has the parent ion [M – H]^−^ at *m/z* 1219.5854 and four main fragment ions at *m/z* 1057 (−162 Da), 1039 (−162 – 18 Da), 925 (−162 – 132 Da) and 587 (−162 – 132 – 162 – 176 Da). The information was consistent with the structure of theasaponin A_4_ (21-*O*-angeloyl-theasapogenol A 3-*O*-β-d-galactopyranosyl-(1→2)-[β-d-glucopyranosyl-(1→2)-α-l-arabinopyranosyl-(1→3)]-β-d-glucuronopyranoside) [29], which has the corresponding fragments including [M – H – glucopyranosyl]^−^, [M – H – glucopyranosyl – H_2_O]^−^, [M – H – glucopyranosyl – arabinopyranosyl]^−^ and [M – H – glucopyranosyl – arabinopyranosyl – galactopyranosyl – glucuronopyranosyl]^−^. In the same way, the peaks 5, 21, 37, 43, 45, 50, and 51 were tentatively identified as theasaponin A_1_ [30], theasaponin A_7_ [31], floratheasaponin A [33], foliatheasaponin I/III [31,35], teaseedsaponin G, teaseedsaponin I, and teaseedsaponin J/K [32], respectively. In addition, the peak 18 was identified as theasaponin E_1_ by comparing the retention time and MS/MS spectra with standard theasaponin E_1_.

**Figure 2:**
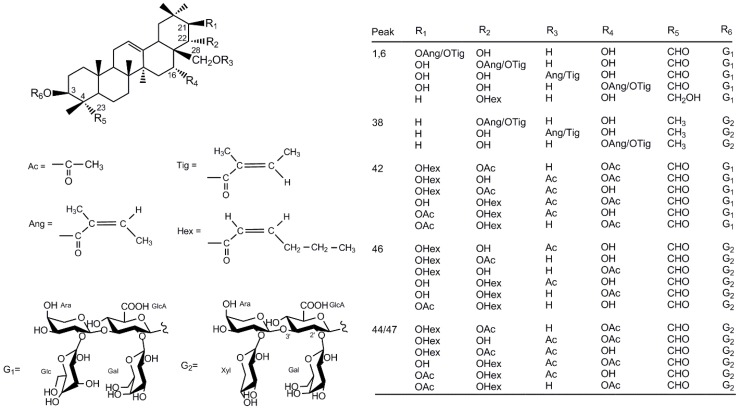
Possible chemical structures of the six potentially new saponins from the *Camellia sinensis* seeds. Ac = acetyl, Ang = angeloyl, Tig = tigloyl, Hex = hexenoyl, Ara = arabinopyranosyl, Gal = galactopyranosyl, Glc = glucopyranosyl, GlcA = glucuronopyranosyl, Xyl = xylopyranosyl.

No saponins with the same molecular weight as the peak 38, 42 and 46 have been reported. Their possible structures were try to speculated based on the known saponins from the seeds of the genus *Camellia*. The peak 38 has parent ion [M – H]^−^ at *m/z* 1157.5841, and fragment ions at *m/z* 1025 (−132 Da), 977 (−162 – 18 Da), 893 (−132 – 132 Da), 875 (−132 – 132 – 18 Da), and 555 (−132 – 132 – 162 – 176 Da). These data suggested that the molecular weight of its sapogenin was around 556, and the sugar moiety contained two pentoses, one hexose, and one hexosuronic acid. Among all the reported saponins from the *Camellia* seeds, only the sapogenin of oleiferasaponin D_1_ in the seeds of *Camellia oleifera* [37], namely 22-*O*-angeloyl-camelliagenin A, has the corresponding molecular weight. The other structure possibilities of the sapogenin results from the angeloyl-linked positions at C-16 and C-28, and the tigloyl is another possible acyl group. The MS/MS fragmentation pattern of peak 38 were consistent with the sugar chain of 38 known saponins from *C. sinensis* seeds, including theasaponin A_1_–A_3_ [30], A_8_, A_9_ [12], B_5_ [31], C_1_ [29], E_1_–E_10_ [26,29,33,36], F_1_–F_3_ [30], G_1_, H_1_ [29], G_2_ [36], assamsaponin A–E [9], teaseedsaponin C, E–G, J–L [35], and floratheasaponin A, I, III [31,33,35], and the oligosaccharidic moiety of all the reported *Camellia* seed saponins is located at C-3. So the compound 38 was tentatively identified as 16/22/28-*O*-angeloyl/tigloyl-camelliagenin A 3-*O*-β-d-galactopyranosyl-(1→2)-[β-d-xylopyranosyl-(1→2)-α-l-arabinopyranosyl-(1→3)]-β-d- glucuronopyranosyl. The peak 42 showed parent ion [M – H]^−^ at *m/z* 1315.6046, and the fragment ions at *m/z* 1153 (−162 Da), 1135 (−162 – 18 Da), 1021 (−162− 132 Da), 1003 (−162 – 132 – 18 Da), 859 (−162 – 132 – 162 Da), and 683 (−162– 132− 162− 176 Da). Its sapogenin may be the similar with that of teaseedsaponin L, namely 16, 22-di-*O*-acetyl-21-*O*-hexenoyl-theasapogenol E [35]. The hexenoyl usually occurs at C-21 or C-22 of the known *Camellia* seed saponins, and the acetyl group may link to the hydroxyl at positions 16, 21, 22, and 28. Its sugar chain possibly corresponded to -β-d-galactopyranosyl-(1→2)-[β-d-glucopyranosyl-(1→2)-α-l-arabinopyranosyl-(1→3)]-β-d-glucuronopyranosyl, which was the oligosaccharidic moiety of theasaponin A_4_–A_7_ [31], E_11_, E_12_ [36], assamsaponin F–I [10], teaseedsaponin A, B, D, H, I [32], and camelliasaponin B_1_, C_1_ [33] in *C. sinensis* seeds. The peak 46 has parent ion [M – H]^−^ at *m/z* 1243.5835, which corresponded to the sapogenin of teaseedsaponin H, 21-*O*-hexenoyl-28-*O*-acetyl-theasapogenol E [32]. Other structure possibilities of its sapogenin result from the positions of hexenoyl and acetyl as peak 42. The fragment ions occurred at *m/z* 1111 (−132 Da), 979 (−132 – 132 Da), 961 (−132 – 132 – 18 Da), and 641 (−132 – 132 – 162 – 176 Da), suggesting its sugar chain probably was -β-d-galactopyranosyl-(1→2)-[β-d-xylopyranosyl-(1→2)-α-l-arabinopyranosyl-(1→3)]-β-d-glucuronopyranosyl as peak 38. The speculated chemical structures of compounds 38, 42, 46 and another three unknown saponins (1, 6, 44, or 47) mentioned were shown in Figure 2. The six compounds were firstly found in the *C. sinensis* seeds, and their structures should be further confirmed by NMR spectra in the future work.

For five groups with ion [M – H]^−^ at *m/z* 1173.5783–1173.5809 (peaks 15, 19), *m/z* 1231.5838–1231.5873 (peaks 11, 14, 22), *m/z* 1261.5947–1261.5989 (peaks 9, 25), *m/z* 1273.5938–1273.5993 (peaks 17, 23, 32, 41), and *m/z* 1301.5922–1301.6239 (peaks 26, 35, 48), the amount of compounds in each group was equal to the number of known *C. sinensis* seed saponins with the similar molecular weight and corresponding fragments. In order to predict the elution order of isomers in each group, Log P value was calculated in SciFinder by launching a chemical structure from ChemBioDraw Ultra 14. Log P is defined as a logarithm of the distribution coefficient of the compound in the liquid/liquid extraction system of water and *n*-octanol. It provides direct information on hydrophobicity, and is frequently used to predict water solubility, soil (sediment)/water partition coefficient, absorption, distribution, metabolism, excretion, and toxicity (ADMET). The higher the Log P value, the more the compound is hydrophobic [38,39]. The group composed of peaks 15 and 19 was taken as an example to elucidate the identification of saponins with a similar molecular weight. The two peaks showed almost the same pseudomolecular ion [M – H]^−^ at *m/z* 1173.58, and similar fragment ions at *m/z* 1041, *m/z* 1023, *m/z* 909, *m/z* 891, *m/z* 747, *m/z* 729, *m/z* 711, and *m/z* 571, which were consistent with theasaponin C_1_ [29] and theasaponin B_5_ [31]. The two compounds may have different sapogenins and the same oligosaccharidic moiety, and are undistinguishable from each other by MS/MS spectra. The Log P values of theasaponin C_1_ and B_5_ were estimated to be 2.0 ± 0.9 and 2.4 ± 0.9, respectively, suggesting that the former saponin was more hydrophilic, and should be eluted earlier than the latter one. So the peak 15 was tentatively determined as theasaponin C_1,_ and the peak 19 was tentatively determined as theasaponin B_5_. Similarly, another four groups of isomers with ion [M – H]^−^ at *m/z* 1231.5838–1231.5873, *m/z* 1261.5947–1261.5989, *m/z* 1273.5938–1273.5993, and *m/z* 1301.5922–1301.6239 were tentatively identified as assamsaponin D (peak 11) [9], theasaponinA_9_ (peak 14) [12], theasaponin A_2_ (peak 22) [30], theasaponin A_6_ (peak 9) [31], theasaponin A_5_ (peak 25) [29], theasaponin A_3_ (peak 17) [30], theasaponin E_10_ (peak 23) [36], theasaponin A_8_ (peak 32) [12], teaseedsaponin F (peak 41) [32], assamsaponin F (peak 26) [10], teaseedsaponin D (peak 35), [35] and theasaponin E_11_ (peak 48) [36].

For the other eight groups with ion [M – H]^−^ at *m/z* 1171.5630–1171.5670 (peaks 7, 28), *m/z* 1187.5587–1187.5953 (peaks 4, 8, 36, 40), *m/z* 1201.5759–1201.5779 (peaks 24, 33), *m/z* 1217.5686–1217.5693 (peaks 1, 6, 10), *m/z* 1229.5699–1229.5725 (peaks 2, 13, 18, 27, 31), *m/z* 1259.5807–1259.5844 (peaks 12, 16, 20, 29, 34), *m/z* 1271.5805–1271.6146 (peaks 30, 39, 49), and *m/z* 1285.5945–1285.5987 (peaks 44, 47), the amount of compounds in each group did not match to the number of known *C. sinensis* seed saponins with the corresponding structures, and/or the Log P values of some isomers were the same. So saponins in the eight groups could only be partly identified according to the MS/MS spectra and the Log P values. The groups with ion [M – H]^−^ at *m/z* 1187.5587–1187.5953 (peaks 4, 8, 36, 40), *m/z* 1217.5686–1217.5693 (peaks 1, 6, 10) and *m/z* 1285.5945–1285.5987 (peaks 44, 47) were discussed here as examples to illustrate the identification of these saponins.

For the group with [M – H]^−^ at *m/z* 1187.5587–1187.5953 (peaks 4, 8, 36, 40), the three peaks 4, 8 and 40 had the similar fragments at *m/z* 1055 (−132 Da), *m/z* 1037 (−132 – 18 Da), *m/z* 923(−132 – 132 Da), *m/z* 905(−132 – 132− 18 Da), *m/z* 761(−132 – 132 – 162 Da), *m/z* 585 (−132 – 132 – 162 – 176 Da). These data were consistent with the structures of theasaponin E_3_, theasaponin E_6_ [33] and teaseedsaponin C [32] isolated from *C. sinensis* seeds, which had the corresponding fragments including [M – H – arabinopyranosyl]^−^, [M – H – arabinopyranosyl – H_2_O]^−^, [M – H – arabinopyranosyl – xylopyranosyl]^−^, [M – H – arabinopyranosyl – xylopyranosyl – H_2_O]^−^, [M – H – arabinopyranosyl – xylopyranosyl – galactopyranosyl]^−^ and [M – H – arabinopyranosyl – xylopyranosyl – galactopyranosyl – glucuronopyranosyl]^−^. The calculated molecular weight of teaseedsaponin C is 0.043 higher than that of theasaponin E_3_ and theasaponin E_6._ In addition, the Log P value of theasaponin E_3_ and E_6_ were both 1.0, while the value of teaseedsaponin C was 2.6. So the Peak 4 and 8 were tentatively determined as either theasaponin E_3_ or E_6_, and the peak 40 was teaseedsaponin C. The peak 36 possesses fragment ions at m/z 1025 (−162 Da), m/z 1007 (−162 – 18 Da), m/z 893 (−162 – 132 Da), m/z 875(−162 – 132 – 18 Da) and m/z 554 (−162 – 132 – 162 – 176 Da), suggesting its sapogenin and sugar chain are different from that of peaks 4, 8 and 40. This compound has not been found in *C. sinensis* seeds before, and its mass spectrometry data is in accordance with camelliasaponin A_1_ and A_2_ from *C. japonica* seeds [34].

Peak 1, 6 and 10 yielded pseudo-molecular ion [M – H]^−^ at *m/z* 1217.5686–1217.5693. Theasaponin F_1_ is the unique known saponin with the corresponding molecular weight from *C. sinensis* seeds [30]. The peak 10 had fragment ions at *m/z* 1085, *m/z* 1067, *m/z* 953, *m/z* 935, m/z 791 and m/z 615, which was corresponded to the fragments of theasaponin F_1_ including [M – H – xylopyranosyl]^−^, [M – H – xylopyranosyl – H_2_O]^−^, [M – H – xylopyranosyl – arabinopyranosyl]^−^, [M – H – xylopyranosyl – arabinopyranosyl – H_2_O]^−^, [M – H – xylopyranosyl – arabinopyranosyl – galactopyranosyl]^−^ and [M – H – xylopyranosyl – arabinopyranosyl – galactopyranosyl – glucuronopyranosyl]^−^, respectively. The peaks 1 and 6 showed the fragment ions at *m/z* 1055 (−162 Da), *m/z* 1037 (−162 – 18 Da), *m/z* 923 (−162 – 132 Da), *m/z* 905 (−162 – 132 – 18 Da), *m/z* 887 (−162 – 132 – 18 – 18 Da), *m/z* 761 (−162 – 132 – 162 Da), and *m/z* 585 (−162 – 132 – 162 – 176 Da). The information indicated that the sapogenins of peak 1 and 6 might be similar with that of theasaponin E_3_, E_6_, and teaseedsaponin C, namely 21-*O*-angeloyl-theasapogenol E and 22-*O*-hexenoyl-theasapogenol C [32,35]. Other structure probabilities of the sapogenins resulted from the presence of angeloyl and tigloyl located at C-16, C-21, C-22, and C-28 of theasapogenol E. There was much chance that their oligosaccharidic moiety were -β-D-galactopyranosyl-(1→2)-[β-d-glucopyranosyl-(1→2)-α-l-arabinopyranosyl-(1→3)]-β-d-glucuronopyranosyl as that of Compound 42 (Figure 2). The two compounds 1 and 6 were firstly found in *C. sinensis* seeds.

Peak 44 and 47 have the pseudo-molecular ion [M – H]^−^ at *m/z* 1285.5945 and *m/z* 1285.5987, and the fragment ions at *m/z* 1153 (−132 Da), *m/z* 1135 (−132 – 18 Da), *m/z* 1021(−132 – 132 Da), *m/z* 1003 (−132 – 132 – 18 Da), *m/z* 859 (−132 – 132 – 162 Da), and *m/z* 683 (−132 – 132 – 162 – 176 Da). Among all the reported *C. sinensis* seed saponins, only teaseedsaponin L corresponds to these data [35]. So either of the peaks 44 and 47 might be teaseedsaponin L, and the another one (probably unreported) in the seeds of *C. sinensis.* Compared with teaseedsaponin L, the potentially-new one might have the diverse substitutions including hexenoyl and acetyl at C-16, C-21, C-22, and C-28 of the sapogenin, and possess the same oligosaccharidic moiety, -β-d-galactopyranosyl-(1→2)-[β-d-xylopyranosyl-(1→2)-α-l-arabinopyranosyl-(1→3)]-β-d-glucuronopyranosyl (Figure 2).

In the same way, the other five groups with ion [M – H]^−^ at *m/z* 1171.5630–1171.5670, *m/z* 1201.5759–1201.5779, *m/z* 1229.5699–1229.5725, *m/z* 1259.5807–1259.5844, and *m/z* 1271.5805–1271.6146 were identified referencing the reported saponins isolated from *C. sinensis* seeds. The retention time, MS/MS data, and Log P values of saponins detected in *C. sinensis* seeds are summarized in Table 1. It was noticed that the elution order of the 51 peaks were not all in the sequence of computer-calculated Log P values. Although Log P is widely used in many quantitative structure-activity relationship (QSAR) models to estimate critical physico-chemical properties of compounds, most of these models exhibit only modest predictive ability due to the chemical diversity of the systems modeled [40]. In this work, Log P value only was used as a reference basis to predict the elution order of isomers. However, it should be noticed that these speculated results need to be further verified due to the limitation of Log P value and the different conformation of isomers which can influence the retention time. Completely-accurate identification results should be obtained depending on the availability of all appropriate standards, or development of efficient on-line hyphenated techniques coupling separation and structural identification. The liquid chromatography (LC)-solid-phase extraction (SPE)-nuclear magnetic resonance (NMR)-mass spectrometry (MS) coupling can greatly simplify the structure identification of unknown compounds in the complex mixtures, without the need for purifying of the analytes [41]. This technique has become a valuable tool for pharmaceutical and natural product research, and is worth being investigated for the analysis of *C. sinensis* seed saponins in the future. Although this UPLC-QTOF-MS/MS method could only provide inference of the compound structures, it was the first time to establish a liquid chromatography method for simultaneously detecting 51 saponins from the *C. sinensis* seeds.

### 2.3. Quantitation of Saponins by UPLC-PDA

Although several chromatographic techniques have been developed for saponin analysis, LC with UV detection remains the routine method for the quality control of saponin in herbal products [18]. In this work, the saponins in *C. sinensis* seeds were quantified by their response to PDA, and theasaponin E_1_ was used as a standard. The calibration curves were linear over the concentration range of 0–1000 μg/mL for theasaponin E_1._ The linear regression equation was Y = 8233.9X + 43043, where X is the concentration and Y is the peak area. The 95% confidence intervals for the regression slope and y-intercept were calculated to range between 8032.5–8435.3 and −62879.8–148965.6. The high correlation coefficient value (R^2^ = 0.9994) indicated a good correlation between standard concentrations and their peak areas within the test ranges. The LLOD (S/N = 3) and LLOQ (S/N = 10) of theasaponin E_1_ were 0.0313 μg/mL and 0.1043 μg/mL, respectively. The intra- and inter-day of accuracy and precision data at three concentrations of theasaponin E_1_ were summarized in Table 2. The accuracy for theasaponin E_1_ ranged from −0.092% to 0.079%, and the precision was between 0.013% and 0.152%. These results met the Guidance for Industry: Bioanalytical Method Validation (2013) proffered by the US Food and Drug Administration (FDA), which recommended the mean value should be within 15% of the nominal value for accuracy, and the precision should not exceed 15% of coefficient of variation (CV). The developed UPLC-PDA method was applied to analyze 70% methanol extract and the total saponin fraction of *C. sinensis* seeds. The individual saponins were quantified, and the total saponin content of the crude extract and the total saponin fraction were calculated to be 19.57 ± 0.05% (wt %) and 41.68 ± 0.09% (wt %), respectively (Table 3). The disadvantage of this method was that all saponin compounds were quantified by the same one standard substance, not by each their own standards. This is due to no commercial *C. sinensis* seed saponin compounds are available, and purify of all these saponins as standards is still difficult so far. In view of the structure similarity of these compounds, this UPLC-PDA method is still relative reliable for quantitation of *C. sinensis* seed saponins.

### 2.4. Comparison between Vanillin-Sulfuric Acid Assay and UPLC-PDA

**Figure 3:**
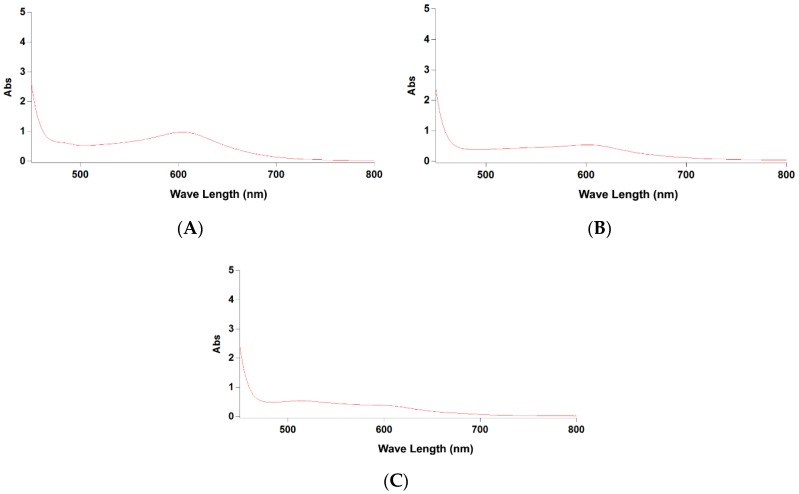
UV-Vis spectra of reaction mixture from vanillin-sulfuric acid assay between 450 nm and 800 nm. (**A**) Theasaponin E_1_ (1.0 mg/mL). (**B**) Total saponin fraction of *C. sinensis* seeds (1.0 mg/mL). (**C**) Crude extract of *C. sinensis* seeds (1.0 mg/mL).

Considering the vanillin-sulfuric acid assay is a classical and popular spectrophotometric method for quantifying saponins from various plant materials [18], the developed UPLC-PDA method was compared with vanillin-sulfuric acid assay in the present work. *C. oleifera* seed saponins have been determined by this colorimetric method, and the detection wavelength was set at 538 nm [19,20]. However, the maximum absorption of reaction mixtures for theasaponin E_1_, 70% methanol extract and the total saponin fraction of *C. sinensis* seeds were found at 600 nm (Figure 3). The standard curve for theasaponin E_1_ was Y = 1.166X – 0.1221 (R^2^ = 0.9982), where X was the concentration and Y was the absorbance. The 95% confidence intervals for the slope and y-intercept were estimated to be 1.051 to 1.271, and −0.190 to −0.047. The contents of saponins in the 70% methanol extract and the purified total saponin fraction were determined to be 43.11 ± 3.17% (wt %) and 56.60 ± 5.79% (wt %), respectively. These results were significantly higher than those determined by the UPLC-PDA method (*p* < 0.05). The former value was more than twice, and the latter was 1.36 folds as compared with the UPLC-PDA data (*p* < 0.05).

Differences in selection of reagent, condition of full color development, standard, and wavelength make it hard to compare the results from different studies using this spectrophotometric assay [18]. More importantly, the vanillin-acid reagent could give a color reaction under acidic conditions with tannins [42], flavonoids [43], proanthocyanidins [44], and steroids [45], thus provide nonspecific and misleading information [21]. As shown in Figure 3, the absorption peaks of the total saponin fraction and crude extract were not obvious, indicating the absorption value might be easily interfered by other impurities. Although several trace saponins were not detected in the crude extract and purified saponin fraction (2 mg/mL) by UPLC-PDA, we thought that the exorbitant detection result from vanillin-sulfuric acid was mainly caused by some interfering substances such as flavonoids. Given the convenience and low cost, the vanillin-sulfuric acid assay could be used to quantify purified *C. sinensis* seed saponins, and give the comparable result with the UPLC-PDA method. However, it is not suitable for determining the saponin content in the crude extract of *C. sinensis* seeds.

## 3. Materials and Methods

### 3.1. Plant Materials and Chemicals

The full ripe fruits of tea (*Camellia sinensis* (L.) O. Kuntze, Theaceae) variety Fudingdabai were harvested from the Tea Resource Garden of China National Tea Museum (Hangzhou, China). The color of these fruits was brown, and their diameter was between 2.7 cm and 3.3 cm. Methanol and acetonitrile of HPLC grade were purchased from Tianjin Shield Company (Tianjin, China). Ethanol, methanol, formic acid, and vanillin of analytical grade were obtained from the National Medicine Chemical Reagent Co. Ltd. (Shanghai, China). The ultrapure water was prepared by a Milli-Q water purification system (Millipore, Bedford, MA, USA).

### 3.2. Preparation of Tea Seed Extract, Total Saponin Fraction and Standard Substance (Theasaponin E_1_)

Tea seeds were removed from shells, freeze-dried and milled into powder by a pulverizer. Then, 800 g powder was extracted under reflux with 12 L of 70% methanol aqueous solution at 70 °C for 5 h. The filtered decoction was concentrated by rotary evaporation, and then lyophilized by a freeze dryer (LGJ-10C, Beijing Sihuan Science Instrument Factory, Bejing, China). The yield of extract from tea seeds was 106.06 g (13.26%).

The crude tea seed extract was suspended in water and extracted successively with petroleum ether, ethyl acetate (EtOAc), and 1-butanol (*n*-BuOH) thrice, respectively. The *n*-BuOH fraction was concentrated under reduced pressure, subjected to D101 column chromatography, and then eluted consecutively with H_2_O, 30%, 50%, and 70% ethanol-aqueous solution (*v/v*) at the flow rate of 3 mL/min each for 2 bed volume (BV, 2.5 L). The 30%, 50%, and 70% ethanol-eluted fractions were collected, concentrated, and lyophilized to yield 9.04 g, 24.69 g and 3.09 g, respectively. Each fraction was detected by the UPLC-PDA-MS method as described in the Section 2.3. The 50% ethanol-eluted fraction had the similar saponin compositions with the crude tea seed extract, and was used as the total saponin fraction for the next work.

**Figure A1:**
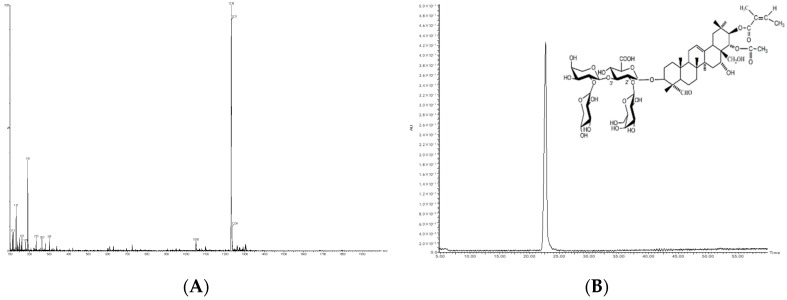
UPLC-PDA-MS chromatogram of theasaponin E_1_ prepared in this work. (**A**) UPLC-UV chromatogram detected at 210 nm and chemical structure of theasaponin E_1_. (**B**) MS spectra of theasaponin E_1_ in negative ion mode.

Theasaponin E_1_ (21-*O*-angeloyl-22-*O*-acetyltheasapogenol E 3-*O*-β-d-galactopyranosyl-(1→2)-[β-d-xylopyranosyl-(1→2)-α-l-arabinopyranosyl-(1→3)]-β-d-glucopyranosiduronic acid) was prepared using a method developed in our lab. Briefly, the total saponin fraction was purified by reversed-phase preparative HPLC system (GE ÄKTA purifier100, Uppsala, Sweden) equipped with a SinoChrom ODS-BP column (5 μm, 250 mm × 10.0 mm i.d., Elite, Dalian, China). Elution was performed with acetonitrile/H_2_O/formic acid (50.0:49.9:0.1, *v/v/v*) at the wavelength of 210 nm and a flow rate of 2.0 mL/min to afford three fractions. The second fraction was further purified on a Waters XBridge Shield RP18 column (5 μm, 250 mm × 10.0 mm i.d., Waters, Milford, MA, USA) to yield theasaponin E1. The mobile phase A was formic acid/water (0.1:99.9, *v/v*), and B was formic acid/acetonitrile (0.1:99.9, *v/v*). The elution gradient was 38% B from 0 to 15 min, then increased to 40% B and maintained for 35 min. The flow rate of the mobile phase was kept at 1.5 mL/min. The purity of the isolated theasaponin E_1_ was determined to be 98% based on the percentage of peak area detected by UPLC-PDA-MS (Figure A1). ^1^H- and ^13^C-NMR spectra were recorded on a Bruker AV-400 (Karlsruhe, Germany) at 500 and 125 MHz, respectively (Table A1). The spectral data (MS, ^1^H- and ^13^C-NMR) of the obtained theasaponin E_1_ were consistent with the published data [26].

### 3.3. Separation and Identification of Saponins by UPLC-PDA-QTOF-MS/MS

The chromatographic separation was performed on an Acquity UPLC instrument equipped with a PDA detector (Waters, Milford, MA, USA) and a Waters Acquity UPLC HSS T3 column (1.8 μm, 150 mm × 2.1 mm i.d., Waters, Milford, MA, USA). The PDA detector was set to scan a range from 200 nm to 400 nm, and the specific wavelength of 210 nm was selected for detecting saponins. The mobile phase A was water containing 0.1% formic acid, and B was acetonitrile containing 0.1% formic acid. Gradient elution was performed as follows: 0–4 min, 35–37% B; 4–32 min, 37% B; 32–58 min, 37–45% B; 58–60 min, 35% B. The sample injection volume was 5 μL, the column was kept at 30 °C, and the flow rate was 0.2 mL/min.

The mass spectrometry analysis was carried out using a hybrid triple quadrupole time-of-flight mass spectrometer (Triple TOFTM 5600 plus System, AB SCIEX, Foster City, CA, USA) equipped with an electrospray ionization (ESI) source in negative ionization mode. The mass range was set at *m/z* 100–2000 for both Time-of-Flight (TOF)-MS and TOF-MS/MS scan. Nitrogen was used as the nebulizer and auxiliary gas. The optimized MS conditions were as follows: ion spray voltage, −4.5 kV; ion source temperature, 500 °C; nebulizing gas (Gas 1), 50 psi; Tis gas (Gas 2), 50 psi; curtain gas, 35 psi; declustering potential (DP), 100 V; collision energy (CE), 10 V. For the MS/MS acquisition mode, the sweeping collision energy was set at 60 ± 20 V for collision-induced dissociation (CID). Data acquisition was carried out using Peak View Software 1.2.0.3 software (AB Sciex, Foster City, CA, USA) in IDA (information-dependent acquisition) mode.

### 3.4. Quantification of Saponins by UPLC-PDA

Quantitative analysis of the individual saponins in the tea seeds was conducted using UPLC-PDA as described in Section 2.3. The method was validated according to Guidance for Industry Bioanalytical Method Validation (FDA) [27]. Theasaponin E_1_ was used as a reference standard, and the calibration curve was constructed at seven concentrations in the range of 50–1000 μg/mL. The “intra-day” and “inter-day” accuracy and precision of the method were determined with three concentration levels (60, 100, and 500 μg/mL) of theasaponin E_1_ on the same day (five replicates) or on five different days. The accuracy, expressed as the percent deviation (% DEV), was calculated as (mean measured concentration − nominal concentration/nominal concentration) × 100%. The precision, expressed as the relative standard deviation (RSD), was calculated as (standard deviation/mean measured concentration) × 100% The lower limits of detection (LLOD) and quantification (LLOQ) were defined as analyte concentrations with a signal-to-noise (S/N) ratio of 3 and 10, respectively, by injecting a series of dilute solutions with known concentrations.

### 3.5. Vanillin-Sulfuric Acid Assay

The total saponin content of tea seeds was determined following the procedures described by Chen et al. with a few modifications [19]. Briefly, 0.5 mL of sample solution was sequentially reacted with 0.5 mL of vanillin ethanol solution (8%, *w/v*) and 4 mL of diluted sulfuric acid (72%, *w/v*). After incubation at 60 °C for 10 min, the reaction mixture was cooled in an ice water bath for another 15 min, and scanned from 450 to 800 nm by an UV-VIS spectrophotometer (UV-2550, Shimadzu, Japan). Theasaponin E_1_ (0.2–1.0 mg/mL) was used as a reference standard to establish the calibration curve.

### 3.6. Statistical Analysis

The data were presented as the mean ± standard deviation (SD). Statistical differences between the two sets of data were evaluated using the Student′s t-test, and a *p* value < 0.05 was considered statistically significant. All statistical analysis of data was performed using the SPSS (Statistical Product and Service Solutions) statistics [26].

## 4. Conclusions

A novel UPLC-PDA-QTOF-MS/MS method was developed and validated for tentatively determining 51 triterpene saponins in *C. sinensis* seeds. Based on the retention behavior, fragmentation pattern and previous literatures, six potentially new saponins (1, 6, 38, 42, 46, 44, or 47) were detected. One saponin (36) identified as camelliasaponin A_1_ or A_2_ from the *C. japonica* seeds [34] was found in the *C. sinensis* seeds for the first time. Compared with the vanillin-sulfuric acid assay, the UPLC-PDA method could quantify individual saponins, and provide more reliable quantitative test results for the total saponin content. Its linearity, accuracy and precision were satisfactory under the requirements of the Guidance for Industry: Bioanalytical Method Validation (FDA, 2013). This work offered a possible routine method for the identification and quantification of *C. sinensis* seed saponins, which was of great significance for the future investigations and applications of these compounds. Moreover, this analytical method could be further developed and validated for the quantification of the saponins in different organs of *C. sinensis* and other *Camellia* species such as *C. reticulata*, *C. japonica,* and *C. sasanqua*, etc.

## Figures and Tables

**Table 1:** Saponins detected in the crude extract of *C. sinensis* seeds by UPLC-PDA-QTOF-MS/MS.

[M – H]^−^ (*m/z*)	Peak	Retention Time (min)	Fragments (*m/z*)	Molecular Formula	Trivial Name	Log P	Reference
1157.5841	38	39.056	1025, 977, 893, 875, 695, 555	C_57_H_90_O_24_	New	-	
1171.5630	7	14.180	1039, 1021, 907, 889, 569	C_57_H_88_O_25_	Theasaponin G_1_	2.1 ± 0.9	[9,29,30]
1171.5670	28	28.314	1039, 1021, 907, 889, 569	C_57_H_88_O_25_	Theasaponin G_2_	2.1 ± 0.9	
					Assamsaponin A	2.1 ± 0.9	
					Assamsaponin E	3.6 ± 0.9	
1173.5783	15	19.949	1041, 1023, 909, 891, 711, 571	C_57_H_90_O_25_	Theasaponin C_1_	2.0 ± 0.9	[27,31,32]
1173.5809	19	23.390	1041, 1023, 909, 891, 747, 729, 571	C_57_H_90_O_25_	Theasaponin B_5_	2.4 ± 0.9	
1185.5774	45	47.343	1053, 1035, 921, 903, 759, 741, 583	C_58_H_90_O_25_	Teaseedsaponin G	2.7 ± 0.9	[32]
1187.5589	4	13.008	1055, 1037, 923, 905, 761, 585	C_57_H_88_O_26_	Theasaponin E_3_	1.0 ± 0.9	[33]
1187.5587	8	14.812	1055, 1037, 923, 905, 761, 585	C_57_H_88_O_26_	Theasaponin E_6_	1.0 ± 0.9	
1187.5953	36	37.143	1025, 1007, 893,875, 554	C_58_H_92_O_25_	Camelliasaponin A_1_/A_2_	3.9 ± 0.9	[34]
1187.5916	40	42.869	1055, 923, 905, 585	C_58_H_92_O_25_	Teaseedsaponin C	2.6 ± 0.9	[32]
1189.5742	5	13.495	1057, 1039, 925, 907, 763, 745, 587	C_57_H_90_O_26_	Theasaponin A_1_	0.8 ± 0.9	[30]
1201.5779	24	26.339	1039, 1021, 907, 889, 871, 727, 709, 569	C_58_H_90_O_26_	Camelliasaponin B_1_/B_2_	2.4 ± 1.0	[34]
1201.5759	32	32.865	1039, 1021, 907, 889, 871, 727, 709, 569	C_58_H_90_O_26_	Camelliasaponin B_1_/B_2_	2.4 ± 1.0	
1215.5892	37	38.814	1083, 1065, 951, 933, 789, 611	C_59_H_92_O_26_	Floratheasaponin A	3.2 ± 0.9	[33]
1217.5689	1	12.071	1055, 1037, 905, 887, 761, 585	C_58_H_90_O_27_	New	-	
1217.5686	6	13.628	1055, 1037, 923, 905, 761, 585	C_58_H_90_O_27_	New	-	
1217.5693	10	15.835	1085, 1067, 953, 935, 791, 615	C_58_H_90_O_27_	Theasaponin F_1_	1.4 ± 0.9	[30]
1219.5854	3	12.675	1057, 1039, 925, 587	C_58_H_92_O_27_	Theasaponin A_4_	1.2 ± 1.0	[29]
1229.5697	2	12.314	1097, 965, 947, 627	C_59_H_90_O_27_	Theasaponin E_1_/E_7_	1.7 ± 0.9	[29,33]
1229.5705	13	18.593	1097, 1079, 965, 947, 785, 627	C_59_H_90_O_27_	Theasaponin E_1_/E_7_	1.7 ± 0.9	
1229.5699	18	22.101	1097, 1079, 965, 947, 785, 627	C_59_H_90_O_27_	Theasaponins E_4_/E_8_	2.0 ± 0.9	
1229.5725	27	27.718	1097, 1079, 965, 947, 803, 785, 627	C_59_H_90_O_27_	Theasaponin E_4_/E_8_	2.0 ± 0.9	
1229.5719	31	32.173	1097, 1079, 965, 947, 803, 785, 627	C_59_H_90_O_27_	Theasaponin E_2_	2.0 ± 0.9	
1231.5847	11	16.616	1099, 1081, 967, 949, 931, 629	C_59_H_92_O_27_	Assamsaponin D	1.6 ± 0.9	[9,30,35]
1231.5838	14	19.605	1099, 967, 949, 931, 629	C_59_H_92_O_27_	Theasaponin A_9_	1.8 ± 0.9	
1231.5873	22	24.643	1099, 1081, 967, 949, 629	C_59_H_92_O_27_	Theasaponin A_2_	2.0 ± 0.9	
1243.5835	46	48.808	1111, 1063, 979, 961, 641	C_60_H_92_O_27_	New	-	
1257.5991	43	45.965	1125, 1107, 993, 975, 957, 831, 654	C_61_H_94_O_27_	Foliatheasaponin I/III	3.7 ± 0.9	[31,35]
1259.5820	12	17.234	1097, 1079, 965, 947, 785, 627	C_60_H_92_O_28_	Assamsaponin G	2.0 ± 1.0	[10,36]
1259.5807	16	20.397	1097, 1079, 965, 947, 785, 627	C_60_H_92_O_28_	Theasaponin E_12_	2.0 ± 1.0	
1259.5844	20	23.966	1097, 1079, 965, 947, 785, 627	C_60_H_92_O_28_	Assamsaponin H	2.5 ± 1.0	
1259.5838	29	29.393	1097, 1079, 965, 947, 785, 627	C_60_H_92_O_28_	Assamsaponin I	2.5 ± 1.0	
1259.5824	33	34.359	1127, 1109, 995, 977, 959, 833, 657	C_60_H_92_O_28_ C_60_H_92_O_28_	Theasaponin F_2_ Theasaponin F_3_	2.1 ± 0.9 2.5 ± 0.9	[30]

1261.5947	9	15.604	1099, 1081, 967, 949, 805, 629	C_60_H_94_O_28_	Theasaponin A_6_	2.1 ± 1.0	[29,31]
1261.5989	25	26.645	1099, 1081, 967, 949, 805, 769, 629	C_60_H_94_O_28_	Theasaponin A_5_	2.3 ± 1.0	
1269.6005	51	58.933	1137,1119,1005,987,843,807,667	C_62_H_94_O_27_	Teaseedsaponin J/K	2.8 ± 0.9	[32]
1271.5841	30	29.634	1139, 1121, 1007, 989, 827, 669	C_61_H_92_O_28_	Assamsaponin B	2.3 ± 0.9	[9,29,32,33]
1271.5805	39	39.601	1139, 1121, 1007, 989, 827, 669	C_61_H_92_O_28_	Theasaponin E_5_/E_9_	2.9 ± 0.9	
1271.6146	49	56.398	1139, 1121, 1007, 989, 845, 827, 669	C_62_H_96_O_27_	Teaseedsaponin E	2.7 ± 0.9	
1273.5958	17	21.009	1141, 1009, 991, 811, 671	C_61_H_94_O_28_	Theasaponin A_3_	2.1 ± 0.9	[12,30,32,36]
1273.5993	23	25.786	1141, 1009, 991, 847, 829, 671	C_61_H_94_O_28_	Theasaponin E_10_	2.3 ± 0.9	
1273.5957	34	36.116	1141, 1009, 991, 847, 829, 671	C_61_H_94_O_28_	Theasaponin A_8_	2.8 ± 0.9	
1273.5938	41	43.580	1141, 1009, 991, 847, 829, 671	C_62_H_98_O_27_	Teaseedsaponin F	2.9 ± 0.9	
1285.5945	44	46.664	1153, 1135, 1021, 1003, 683	C_62_H_94_O_28_	Teaseedsaponin L	2.9 ± 0.9	
1285.5987	47	53.821	1153, 1135, 1021, 1003, 859, 683	C_62_H_94_O_28_	New		[32]
1299.6105	50	57.373	1137, 1119, 1005, 987, 667	C_63_H_96_O_28_	Teaseedsaponin I	3.1 ± 1.0	[32]
1301.5942	26	27.364	1139, 1121, 1007, 989, 809, 669	C_62_H_94_O_29_	Assamsaponin F	2.6 ± 1.0	[10,32,36]
1301.5922	35	36.828	1139, 1121, 1007, 989, 845, 809, 669	C_62_H_94_O_29_	Theasaponin E_11_	3.2 ± 1.0	
1301.6239	48	55.001	1139, 1121, 1007, 989, 845, 809, 669	C_63_H_98_O_28_	Teaseedsaponin D	3.0 ± 1.0	
1303.6111	21	24.213	1141, 1123, 1009, 991, 811, 671	C_62_H_96_O_29_	Theasaponin A_7_	2.4 ± 1.0	[31]
1315.6046	42	44.765	1153, 1135, 1021, 1003, 859, 683	C_63_H_96_O_29_	New	-	

**Table 2:** Intra-day and inter-day accuracy and precision of theasaponin E_1_ at three concentrations.

Nominal Concentration (μg/mL)	Calculated Concentration (μg/mL)		Accuracy (DEV, %)		Precision (RSD, %)		95% Confidence Interval	
	Intra-Day	Inter-Day	Intra-Day	Inter-Day	Intra-Day	Inter-Day	Intra-Day	Inter-Day
60	59.952 ± 0.091	59.945 ± 0.012	0.080	0.092	0.152	0.020	59.726–60.178	59.915–59.975
100	100.079 ± 0.095	100.046 ± 0.056	0.079	0.046	0.095	0.056	99.843–100.315	99.907–100.185
500	499.960 ± 0.065	500.013 ± 0.091	0.008	0.003	0.013	0.018	499.799–500.122	499.787–500.239

**Table 3:** Saponin content of the crude extract and total saponin fraction of *C. sinensis* seeds determined by UPLC-PDA.

Peak	Crude Extract		Total Saponin Fraction	
	Retention Time (min)	Content (wt %)	Retention Time (min)	Content (wt %)
1	ND	ND	12.12	0.08 ± 0.01
2				
3	ND	ND	ND	ND
4	12.95	0.13 ± 0.01	12.59	0.31 ± 0.01
5				
6	13.26	0.04 ± 0.01	12.88	0.14 ± 0.01
7	13.72	0.15 ± 0.01	13.29	0.46 ± 0.01
8	14.76	0.49 ± 0.01	14.31	0.99 ± 0.01
9	ND	ND	14.90	0.15 ± 0.01
10	15.77	0.11 ± 0.02	15.34	0.16 ± 0.01
11	16.55	0.14 ± 0.01	16.08	0.17 ± 0.01
12	17.32	0.17 ± 0.01	16.76	0.32 ± 0.01
13	18.34	0.16 ± 0.01	17.77	0.26 ± 0.02
14	18.72	0.83 ± 0.01	18.17	1.04 ± 0.02
15	19.60	1.02 ± 0.01	19.00	1.11 ± 0.02
16	19.99	0.34 ± 0.01	19.39	0.44 ± 0.02
17	20.72	1.07 ± 0.01	20.01	1.60 ± 0.01
18	22.43	5.43 ± 0.02	21.82	7.62 ± 0.02
19	23.42	0.31 ± 0.01	22.77	0.82 ± 0.02
20	23.95	0.76 ± 0.01	23.32	1.14 ± 0.02
21				
22	ND	ND	24.33	0.15 ± 0.01
23	25.81	0.48 ± 0.01	24.97	0.96 ± 0.02
24	26.80	0.42 ± 0.02	25.99	0.98 ± 0.01
25	27.63	0.17 ± 0.01	26.84	0.30 ± 0.02
26	28.36	0.55 ± 0.01	27.47	0.94 ± 0.01
27	28.84	1.65 ± 0.01	28.06	2.94 ± 0.02
28	30.02	0.82 ± 0.02	29.02	2.40 ± 0.01
29				
30	31.19	0.08 ± 0.01	30.10	0.17 ± 0.01
31	32.61	1.18 ± 0.01	31.56	4.93 ± 0.01
32	33.26	0.12 ± 0.02	32.53	0.24 ± 0.01
33	34.58	0.34 ± 0.01	33.47	0.81 ± 0.01
34				
35	36.19	0.15 ± 0.01	35.28	0.27 ± 0.01
36	37.53	0.23 ± 0.01	36.73	0.68 ± 0.02
37	38.85	0.18 ± 0.01	38.01	0.34 ± 0.01
38	39.79	0.64 ± 0.01	38.97	2.06 ± 0.02
39				
40	ND	ND	42.49	0.28 ± 0.01
41	ND	ND	42.80	0.17 ± 0.01
42	ND	ND	44.254	0.23 ± 0.01
43	ND	ND	45.39	0.37 ± 0.01
44	46.93	0.32 ± 0.02	46.21	1.09 ± 0.03
45	47.81	0.17 ± 0.05	47.07	0.92 ± 0.02
46	48.94	0.18 ± 0.01	48.30	0.40 ± 0.01
47	54.09	0.12 ± 0.01	53.30	0.34 ± 0.02
48	ND	ND	54.21	0.31 ± 0.01
49	56.21	0.27 ± 0.01	55.57	0.93 ± 0.01
50	ND	ND	56.68	0.38 ± 0.01
51	58.82	0.35 ± 0.01	58.29	1.28 ± 0.01
Sum		19.57 ± 0.05		41.68 ± 0.09

ND: Not detected.

## Appendix A

**Table A1:** ^13^C- and ^1^H-NMR spectral data of theasaponin E_1_ prepared in this work (pyridine-d5).

Carbon	^13^C-NMR *δ* (ppm)	^1^H-NMR *δ* (ppm), Multiplicity, *J_HH_* (Hz)
C-1	37.91	
C-2	25.00	
C-3	84.28	
C-4	54.91	
C-5	48.08	
C-6	20.12	
C-7	32.15	
C-8	40.03	
C-9	46.51	
C-10	35.78	
C-11	23.52	
C-12	122.75	5.38 (1H), br s
C-13	142.67	
C-14	41.44	
C-15	34.30	
C-16	67.67	
C-17	47.75	
C-18	39.84	
C-19	46.90	
C-20	36.02	
C-21	78.63	6.62 (1H), d, 10.1
C-22	74.11	6.21 (1H), d, 10.1
C-23	209.74	9.93 (1H), s
C-24	10.82	1.48 (3H), s
C-25	15.53	0.81 (3H), s
C-26	16.54	0.87 (3H), s
C-27	27.14	1.78 (3H), s
C-28	63.57	
C-29	29.22	1.10 (3H), s
C-30	20.02	1.22 (3H), s
GlcA-1′	103.91	4.86 (1H), d, 7.3
2′	78.02	
3′	83.78	
4′	70.58	
5′	77.04	
6′	171.89	
Gal-1′′	103.02	5.79 (1H), d, 7.6
2′′	73.47	
3′′	75.15	
4′′	70.27	
5′′	76.32	
6′′	61.82	
Ara-1′′′	101.39	5.77 (1H), d, 6.5
2′′′	82.08	
3′′′	73.12	
4′′′	68.09	
5′′′	65.75	
Xyl-1′′′′	106.82	5.02 (1H), d, 7.6
2′′′′	75.70	
3′′′′	77.99	
4′′′′	70.51	
5′′′′	67.27	
Ang-1′′′′′	167.61	
2′′′′′	128.73	
3′′′′′	136.78	6.00 (1H), dq-like
4′′′′′	15.65	2.11 (3H), d, 7.0
5′′′′′	20.76	2.03 (3H), s
Ac-1′′′′′′	170.72	
2′′′′′′	20.62	1.93 (3H), s

GlcA: β-d-glucopyranosiduronic acid; Gal: β-d-galactopyranosyl; Ara: α-l-arabinopyranosyl; Xyl: β-d-xylopyranosyl; Glc: β-d-glucopyranosyl; Ac: acetyl; Ang: angeloyl.
